# *In vivo *activity of plant-based interleukin-12 in the lung of Balb/c mouse

**DOI:** 10.1186/1756-0500-3-151

**Published:** 2010-05-27

**Authors:** Carla Sánchez-Hernández, Abel Gutiérrez-Ortega, Diana Aguilar-León, Rogelio Hernández-Pando, Miguel Gómez-Lim, Beatriz Gómez-García

**Affiliations:** 1 Centro Universitario de Ciencias Biológicas y Agropecuarias, Universidad de Guadalajara, Zapopan, Jalisco, México; 2 Centro de Investigación y Asistencia en Tecnología y Diseño del Estado de Jalisco A.C., Guadalajara, Jalisco, México; 3 Departamento de Patología, Instituto Nacional de la Nutrición Salvador Zubirán, México, D.F. México; 4 Departamento de Ingeniería Genética, Centro de Investigación y de Estudios Avanzados, Irapuato, México; 5 Departamento de Microbiología y Parasitología, Facultad de Medicina, Universidad Nacional Autónoma de México, México, D.F., México

## Abstract

**Background:**

In the last years, plants are being used for the production of a wide variety of biopharmaceuticals, including cytokines, and have the potential to serve as vehicles for mucosal administration of these molecules. We had previously reported the expression of a cytokine, interleukin-12 (IL-12), in transgenic tomato plants and had demonstrated that it retained its biologic activity *in vitro*.

**Findings:**

In this work, we administered crude extracts of IL-12-containing tomato fruits to mice through the intratracheal route, measuring endogenous IL-12 and determining biologic activity by quantification of interferon-gamma (IFN-γ) in lungs and by histological analysis. IFN-γ expression in lungs, as well as histological analysis, indicate that tomato-expressed IL-12 retains its biologic activity and, most importantly, its effects are restricted to the site of administration.

**Conclusion:**

Our results indicate that the functional activity of tomato-expressed IL-12 is comparable to that of commercial recombinant IL-12 when given via the mucosal route. This opens the possibility of using crude extracts prepared from tomatoes expressing IL-12 for certain immunotherapies.

## Background

Interleukin-12 (IL-12), a member of the heterodimeric cytokine family, is a pro-inflammatory cytokine that favours the differentiation of T helper 1 cells, promoting cellular immune responses. The therapeutic potential of IL-12 has been extensively examined using a variety of animal models *in vivo*. IL-12 augments immune responses to a variety of infectious agents known to be controlled via cellular immunity, elicits significant antitumor effects and, there is promising data supporting the use of IL-12 as a vaccine adjuvant [1].

The limited use of IL-12 as a therapeutic agent is mainly due to the fact that subcutaneous administration, which is the route most common employed, is associated with highly elevated levels of serum interferon-γ (IFN-γ) that lead to significant toxicity. For this reason, there is interest in identifying alternative approaches for IL-12 delivery that are efficacious without causing adverse effects. In this regard, it has been demonstrated that mucosal delivery of IL-12 induces much less toxicity than parenteral administration, while maintaining the efficacy [2]. This finding opens the need for suitable vehicles for IL-12 mucosal administration.

Transgenic plants are excellent candidates as vehicles for mucosal administration of therapeutic proteins and offer some advantages over other expression systems, such as low-cost inputs, feasibility of scaling up, reduction of health risks deriving from contamination with human pathogens, simplification of downstream processing and the ability to perform complex posttranslational modifications [3]. To date, there are several publications reporting the expression of cytokines in plants, including IL-12, that fully retain their activity *in vitro *[4-7]. However, with only one exception, where tobacco extracts expressing IL-10 were administered orally to mice [8], experiments to evaluate *in vivo *activity of plant-based cytokines have not yet been conducted.

In a previous work, we reported the generation of transgenic tomato plants that express single-chain mouse IL-12 and showed that this plant recombinant protein displayed biologic activity *in vitro*, as determined by IFN-γ production by T lymphocytes after the addition of transgenic plant crude extracts [9]. In the present study, we examined tomato-expressed IL-12 for its *in vivo *activity, by delivering fruit crude extracts intratracheally to mice. IFN-γ expression in lungs, as well as histological analysis, indicate that tomato-expressed IL-12 retains its biologic activity and, most importantly, its effects are restricted to the site of administration.

## Results and Discussion

Here we describe the *in vivo *activity of a single-chain IL-12 expressed in an edible crop, the tomato. At present, the crop that has been used the most for production of immunomodulatory molecules is tobacco. This represents, however, an important disadvantage if the recombinant protein is intended to be administered without purification, because of the presence of alkaloids in tobacco extracts. For this reason, the use of edible crops as factories for biopharmaceuticals is highly desirable, particularly for mucosal administration. One interesting approach is that reported by Menassa et al. [8], where IL-10 was expressed in low alkaloid tobacco plants grown in controlled environment chambers and its oral administration significantly reduced the severity of inflammatory bowel disease in a mouse model.

Since we originally planned to administer recombinant murine IL-12 expressed in tomato fruits (tIL-12) without purification, and considering that when free IL-12 beta chain is found in excess, it has the capacity to form homodimers that function as specific IL-12 antagonists, we decided to express a single-chain IL-12 molecule encoded by only one gene, ensuring this way that there would be equivalent amounts of both IL-12 chains [10]. Due to the high IL-12 levels obtained in our transgenic tomato fruits, as compared to those obtained for other cytokines [4-6], it was possible to administer an adequate intratracheal dose of IL-12 to mice in a small volume.

**Figure 1:**
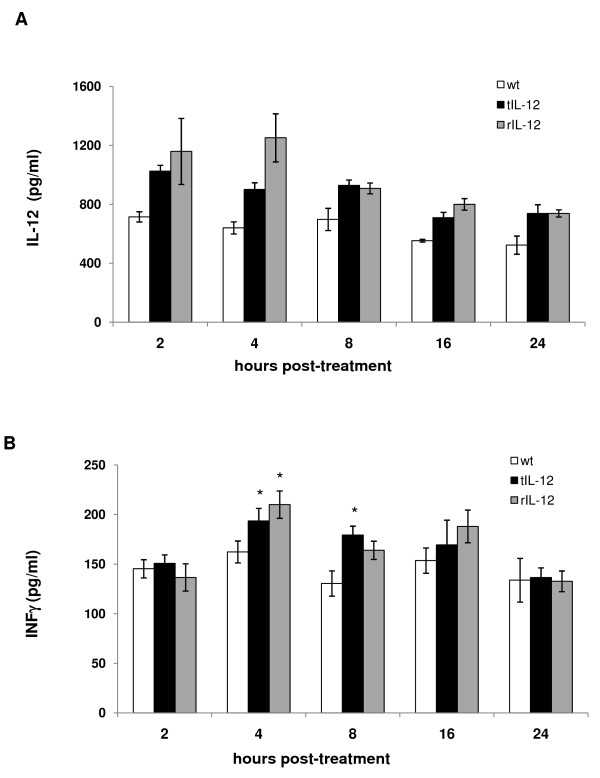
**Changes in IL-12 and INF-γ levels over time in IL-12-treated mice**. BALB/c mice were administered with either tIL-12 or rIL-12 as described in the methods section and sacrificed 2, 4, 8, 16 and 24 h after treatment and their lungs removed. Whole lung homogenates were prepared and IL-12 (A) and INF-γ (B) assayed by ELISA. Mice treated with extracts from a non-transgenic wild type tomato (wt) were included as controls. Results are given as mean ± SEM (n = 4 for each group). * P < 0.05 *vs *control.

The time course of IL-12 levels was analyzed in lung homogenates and serum at 2, 4, 8, 16 and 24 h after IL-12 treatment (tIL-12 and commercial recombinant, rIL-12). IL-12 protein level in lungs was higher in mice treated with IL-12 than that seen in control mice at all evaluated time points (Figure 1A), which indicates that IL-12 was successfully delivered to the lungs. In addition, small differences in IL-12 levels between recombinant and plant IL-12 treatments were observed. These differences may be caused by the presence of proteases in tomato crude extracts that moderately degrade IL-12 before it is absorbed through respiratory epithelium. A 5% of weight loss was observed on both IL-12 treatments by 24 h (data not shown). Hypoglycemia and decreased food intake have been observed previously in mice treated with IL-12 [11], and perhaps this is the explanation for the weight loss in our experiments. The amount of IL-12 in sera was undetectable in all treatments.

Since IL-12 has a half-life of 5 to 10 h [12], the prediction was that exogenous IL-12 would alter INF-γ production early in the lung. As shown in Figure 1B, INF-γ levels increased significantly at 4 and 8 h in mice treated with IL-12; tIL-12 and rIL-12 induced INF-γ levels in a similar way. By 24 h, INF-γ in the lungs of IL-12-treated mice decreased to the level determined in control mice. INF-γ levels were undetectable in mice serum. This demonstrates, on one hand, that both IL-12s had biological activity and, on the other hand, the response was locally confined.

Our results clearly indicate that plant-expressed IL-12, like recombinant IL-12, was successfully delivered to the lungs, as observed by the IL-12 increase in mice treated with tomato fruit crude extracts or recombinant IL-12. Both IL-12s induced the production of comparable IFNγ levels and the response was induced in a local fashion, according to the absence of IL-12 and IFNγ levels in peripheral blood of treated mice. This observation support that of Huber et al. [2], where intranasal IL-12 administration had no effects on serum IFNγ levels, which correlates with IL-12 reduced toxicity. However, a mild toxicity was observed in mice treated with plant-expressed IL-12 as well as recombinant IL-12, according to a slight weight loss in both treatments (data not shown).

**Figure 2:**
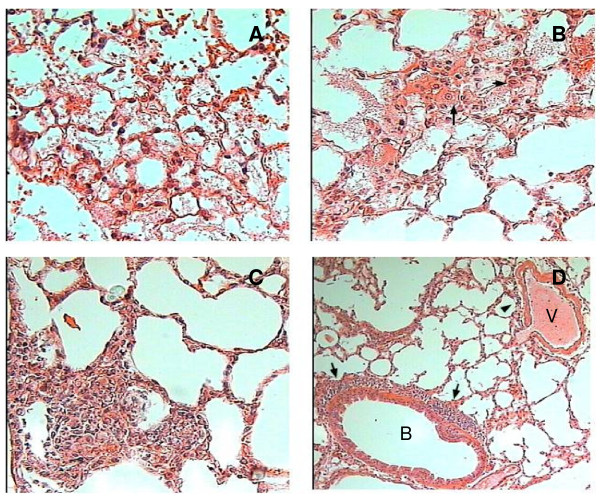
**Representative histological features of lung sections stained with hematoxylin-eosin after intratracheal administration of rIL-12, tIL-12 and non-transgenic wild type tomato extract**. Lung sections 2 h after administration of non-transgenic wild type tomato extracts (A), 12 h after tIL-12 administration (B), where alveoli with fibrilar material mixed with activated macrophages (arrows) and lymphocytes are seen, and 24 h after rIL-12 administration (C, D). Some venules (V) and bronchioles (B) are surrounded by inflammatory cells (arrows).

After 2 h of intratracheal administration of tIL-12 or non-transgenic control extracts, mice lungs showed occasional patches of eosinophilic fibrilar material into alveolar spaces with few neutrophils (Figure 2A), while no significant histological changes were seen in mice that received rIL-12. Two hours later, perivenular inflammation and alveolar spaces with fibrilar material surrounded by neutrophils and macrophages were seen in mice treated with tIL-12 or wt extracts. At 12 h and, more evidently, 24 h after the intratracheal administration of tIL-12, mice lung showed nodular patches of intralveolar inflammation constituted by activated macrophages (large cells with compact cytoplasm and big nucleus with marginated chromatin and apparent nucleoli) surrounded by lymphocytes (Figure 2B). Similar inflammatory infiltrates were seen in the lung of mice that received rIL-12 (Figure 2C); bronchioli and blood vessels were surrounded by inflammatory cells (Figure 2D).

It is important to mention that the results presented here are from a single dose of 100 ng IL-12; nevertheless, we first tested a dose-response relationship between IL-12 administration and IFN-γ production, but no increase in IFN-γ levels was observed when using IL-12 doses higher than 100 ng (data not shown).

In conclusion, we showed in this report that tomato-expressed IL-12 displays a functional activity that is similar to that of recombinant IL-12 *in vivo *when administered through a mucosal route. This opens the possibility of using crude extracts prepared from tomatoes expressing IL-12 for certain immunotherapies where the induction of a Th1 response is necessary. To this respect, the therapeutic effects of plant-based IL-12 were evaluated by oral administration of tomato extracts to mice infected with *Mycobacterium tuberculosis *and it was found that plant-based IL-12 reduced lung mycobacterial loads, as a consequence of an effective induction of a Th1 response by IL-12 [13]. Finally, we are currently testing the efficacy of plant-based IL-12 in the control of human respiratory syncytial virus infection in a mouse model.

## Methods

### Animals

Male BALB/c mice at 6-8 weeks of age were maintained in pathogen-free conditions according to guidelines of the Ethical Committee for Experimentation in Animals of the National Institute of Medical Sciences and Nutrition in Mexico.

### Plant extract preparation

Tomato fruit extracts from line IL12-1, generated by *Agrobacterium*-mediated transformation with binary plasmid p35SMIL12-2300, as reported elsewhere [9], were prepared as follows: 2 volumes of ice-cold extraction buffer (0.01 M Na_2_HPO_4_, 0.003 M KH_2_PO_4_, 0.1 M NaCl and 0.025 M sodium ascorbate) were added to fruit tissue previously ground in liquid nitrogen, crude extracts were centrifuged at 10,000 rpm for 10 min at 4°C and cleared supernatans were filtered through sieve cloth and lyophilized. Lyophilized extracts were reconstituted in 0.2 μM-filtered, deionized water and IL-12 levels were determined by ELISA with a commercial kit (BD Biosciences, San Diego CA).

### IL-12 treatment

Recombinant murine IL-12 expressed in tomato fruits (tIL-12) or commercial recombinant IL-12 (Sigma, St. Louis, MO) (rIL-12) were administered dissolved in PBS by direct intratracheal cannulation in a single dose (100 ng). Control mice were treated with extracts from a non-transgenic plant (wt), using the same amount of protein as in tomato IL-12 extracts. Groups of 4 mice were anaesthetized with sevofluorane vapors (Abbott Laboratories, México) and immobilized on cardboard. Then, tomato extracts or recombinant cytokine were administered using a rigid stainless steel cannula (Thomas Scientific, Swedesboro, NJ) connected to an insulin syringe. The cannula was introduced into the mouth and then directly into the trachea. Mice were maintained in vertical position until the effect of the anesthesia passed. Lung and serum samples were harvested at various times after IL-12 treatment (2, 4, 8, 16 and 24 h). Two independent experiments were performed.

### ELISAs

Whole lung homogenates were prepared using a tissue homogenizer (Kinematica, Luzern, Switzerland) and 1 ml of PBS. Levels of IL-12 and INF-γ were measured using commercial available ELISA kits (BD Biosciences, San Diego CA), following the manufacturer's instructions. The limit of detection of the assay was 62.5 pg/ml of IL-12 and 31.3 pg/ml of INF-γ.

### Histological analysis

Mouse lungs were perfussed with 100% ethanol via the trachea and removed for histological analysis. Paraffin-embedded tissue was sectioned to a thickness of 5 μm and stained with hematoxylin and eosin. Histopathology of various tissues was evaluated in a blinded fashion.

### Statistics

Numerical results were expressed as means ± SEM. Pairs of groups were compared by Student's t-test and significance was determined with *P *values < 0.05.

## Competing interests

The authors declare that they have no competing interests.

## Authors' contributions

CSH prepared extracts from transgenic tomato line expressing mouse IL-12, carried out most of the experimental work and helped in manuscript drafting. AGO developed the transgenic tomato line expressing mouse IL-12, helped in conceiving the experiment and drafted the manuscript. DAL administered tIL-12 and rIL-12 intratacheally to mice and performed histological analysis. RHP participated in experiment design. MGL helped in manuscript drafting and provided plant material. BGG conceived and designed the experiment. All authors read and approved the final manuscript.
